# Preserved skeletal muscle protein anabolic response to acute exercise and protein intake in well-treated rheumatoid arthritis patients

**DOI:** 10.1186/s13075-015-0758-3

**Published:** 2015-09-25

**Authors:** Ulla Ramer Mikkelsen, Kasper Dideriksen, Mads Bisgaard Andersen, Anders Boesen, Nikolai Mølkjær Malmgaard-Clausen, Inge Juul Sørensen, Peter Schjerling, Michael Kjær, Lars Holm

**Affiliations:** Institute of Sports Medicine, Department of Orthopaedic Surgery M, Bispebjerg Hospital and Center for Healthy Aging, Faculty of Health and Medical Sciences, University of Copenhagen, Bispebjerg Hospital bldg 8, Bispebjerg Bakke 23, 2400 Copenhagen NV, Denmark; Section for Sports Science, Institute of Public Health, Aarhus University, Dalgas Avenue 4, 8000 Aarhus C, Denmark; Copenhagen Center for Arthritis Research (COPECARE), Center for Rheumatology and Spine Diseases, Centre of Head and Orthopaedics, Rigshospitalet, Glostrup Hospital, University of Copenhagen, Nordre Ringvej 57, 2600 Glostrup, Denmark; Department of Biomedical Sciences, Faculty of Health and Medical Sciences, University of Copenhagen, Blegdamsvej 3B, 2200 Copenhagen, Denmark

## Abstract

**Introduction:**

Rheumatoid arthritis (RA) is often associated with diminished muscle mass, reflecting an imbalance between protein synthesis and protein breakdown. To investigate the anabolic potential of both exercise and nutritional protein intake we investigated the muscle protein synthesis rate and anabolic signaling response in patients with RA compared to healthy controls.

**Methods:**

Thirteen RA patients (age range 34–84 years; diagnosed for 1–32 years, median 8 years) were individually matched with 13 healthy controls for gender, age, BMI and activity level (CON). Plasma levels of C-reactive protein (CRP), interleukin (IL)-6 and tumor necrosis factor (TNF)-α were measured using enzyme-linked immunosorbent assay (ELISA) in resting blood samples obtained on two separate days. Skeletal muscle myofibrillar and connective tissue protein fractional synthesis rate (FSR) was measured by incorporation of the amino acid ^13^C_6_-phenylalanine tracer in the overnight fasted state for 3 hours (BASAL) and 3 hours after intake of whey protein (0.5 g/kg lean body mass) alone (PROT, 3 hrs) and in combination with knee-extensor exercise (EX) with one leg (8 × 10 reps at 70 % of 1RM; PROT + EX, 3 hrs). Expression of genes related to inflammatory signaling, myogenesis and muscle growth/atrophy were analyzed by real-time reverse transcriptase-polymerase chain reaction (RT-PCR).

**Results:**

CRP was significantly higher in the RA patients (2.25 (0.50) mg/l) than in controls (1.07 (0.25) mg/l; *p* = 0.038) and so was TNF-α (RA 1.18 (0.30) pg/ml vs. CON 0.64 (0.07) pg/ml; *p* = 0.008). Muscle myofibrillar protein synthesis in both RA patients and CON increased in response to PROT and PROT + EX, and even more with PROT + EX (*p* < 0.001), with no difference between groups (*p* > 0.05). The gene expression response was largely similar in RA vs. CON, however, expression of the genes coding for TNF-α, myogenin and HGF1 were more responsive to exercise in RA patients than in CON.

**Conclusions:**

The study demonstrates that muscle protein synthesis rate and muscle gene expression can be stimulated by protein intake alone and in combination with physical exercise in patients with well-treated RA to a similar extent as in healthy individuals. This indicates that moderately inflamed RA patients have maintained their muscle anabolic responsiveness to physical activity and protein intake.

**Electronic supplementary material:**

The online version of this article (doi:10.1186/s13075-015-0758-3) contains supplementary material, which is available to authorized users.

## Introduction

Rheumatoid arthritis (RA) is a systemic, inflammatory, autoimmune disease primarily affecting the joints [1]. Patients with RA are often characterized by having a lower muscle mass than their peers [2] and one of the causal mechanisms has been suggested to be related to the chronic inflammatory state [3]. Rat studies show that the development of low-grade inflammation negatively affects muscle mass and attenuates the muscle protein synthesis response to feeding [4, 5]. Moreover, plasma from cachectic patients (cancer and septic shock), characterized by high levels of inflammatory markers, can induce inflammatory signaling and loss of muscle protein in cultured muscle cells [6–8]. Likewise, an increased level of systemic inflammation may contribute to the muscle loss observed in relation to other diseases like cancer, chronic obstructive pulmonary disease (COPD) and diabetes [8–15]. Evidently, the loss of muscle mass leads to muscle strength deficits and in addition, RA patients may have reduced muscle strength due to greater intramuscular fat infiltration [16] along with pain-related limitations. In addition to the repeatedly reported reduction in muscle strength in RA patients [16–18], metabolic changes occur in both preclinical and later RA stages, including deterioration of blood lipid profile and insulin sensitivity [19–21] which may increase cardiovascular disease risk, summing up to a reduced life span [22]. All of these conditions could be rejuvenated by improving skeletal muscle mass and quality by means of exercise and nutritional interventions, highlighting the importance of understanding the molecular regulation of muscle mass in RA.

Resistance exercise enhances protein turnover rate, thus increases both protein synthesis and breakdown rates. However, a concomitant intake of dietary protein further stimulates muscle protein synthesis resulting in a net protein synthesis and thus protein accretion. When repeated, it makes up a strategy to counteract loss of muscle mass and strength.

In the present study, we aimed to investigate skeletal muscle mass regulation in methotrexate-treated RA patients, measuring leg muscle protein synthesis and expression of genes involved in myogenesis, inflammatory signaling and growth/atrophy in response to resistance exercise and whey protein supplementation in RA patients compared with that of control subjects. Each RA patient was carefully matched with a control subject based on age, gender, activity level and body mass index (BMI) to rule out direct effects of these parameters and focus on effects specifically related to the RA disease. The age and activity matching was important, since an impaired anabolic response - anabolic resistance - is reported with aging [23], and as even within the normal range of inflammatory indicators, both age and level of physical activity plays a role and could contribute to reductions in muscle mass with RA.

In a rat model of RA, adjuvant-induced arthritis, remarkable changes in skeletal muscle have been demonstrated [24–30], including increased mRNA expression of tumor necrosis factor (TNF)-α, muscle ring-finger protein (MuRF1), atrogin1, insulin-like growth factor (IGF)-1, MyoD and myogenin in relation to muscle wasting and a reduced body weight gain during growth. Similar alterations in RA patients may underlie the muscle deteriorations observed in these patients. However, whether expression of such anabolic or proteolytic pathway genes is altered in muscle of RA patients and how these are regulated by exercise and protein intake is to our knowledge currently unknown.

In recent years, treatment of RA patients has improved resulting in better quality of life for most patients. Therefore, we included well-treated RA patients receiving the anchor disease-modifying antirheumatic drug (DMARD), methotrexate, which is first-line medical treatment for RA patients. Patients receiving biological anti-TNF-α or steroid therapy were excluded, in order to obtain a homogenous experimental group, and due to the fact that glucocorticoids are expected to markedly affect skeletal muscle per se [31]. Although the level of systemic inflammation in RA is somewhat reduced during antirheumatic treatment, it has been shown not to lower the level completely down to that of healthy peers [32, 33], and we anticipated that the well-treated RA patients included in this study would still have increased levels of systemic inflammation [17, 32, 34–38].

This study reports for the first time an anabolic response of myofibrillar and collagen protein synthesis and gene expression to acute resistance exercise and protein feeding in RA patients, similar to that of healthy controls. However, expression of the genes coding for TNF-α, myogenin and hepatocyte growth factor (HGF)1 were more responsive in RA patients compared to controls.

## Methods

### Subjects

Thirteen patients diagnosed with RA according to the American College of Rheumatology (ACR) classification criteria from 1987 and not receiving anti-TNF-α or steroid therapy (6 months and 6 weeks washout, respectively) were included. All patients received methotrexate. Time since RA diagnosis ranged from 1 to 32 years (median 8 years). Exclusion criteria were; type 2 diabetes, BMI above 38, cardiovascular disease, cancer or known infections. Disease Activity Score in 28 joints (DAS28) range was 1.8–4.6 (mean 2.6, SD 1.0, n = 6), of these 50 % were seropositive. Each patient (RA) was carefully matched with a healthy control subject (CON) based on gender, age (+/− 2 yrs) and BMI (+/− 2 units). RA patients were classified into one of four groups (1–4) of physical activity level according to the physical activity part of the Copenhagen City Heart Study questionnaire (Østerbroundersøgelsen, [39]). Matching CON subjects had to fit into the same activity group as the corresponding RA patient. For inclusion of matching controls 150 candidates were screened by telephone interviews. The study was approved by the Research Ethics Committees of the Capital Region of Denmark (H-4-2011-028) and conformed to the Declaration of Helsinki. All subjects gave written informed consent before participation. Subjects were asked to refrain from caffeine and alcohol for 1 and 3 days, respectively, prior to the experiment and to avoid exercise for the last 2 days before the experimental day.

### Pretest day

Prior to the experimental day, subjects met for a pretest day for anthropometric measures, strength tests, dual-energy X-ray absorption (DXA) scanning, blood sampling, interview etc. Height was measured to the nearest centimeter and weight to the nearest 100 g, wearing light clothes and without shoes. Waist circumference was measured as the smallest circumference between anterior superior iliac spine and the lower ribs, and hip circumference as the largest circumference around the hips, both to the nearest centimeter. Body mass index (BMI) and waist/height ratio were calculated from these measurements. Following 10 min of supine rest, blood pressure and ‘resting’ heart rate were measured (Table 1a). Physical activity level of included subjects was recorded by use of the Physical Activity Scale (PAS) and converted to metabolic equivalent of task (MET) values as described by Aadahl and Jorgensen [40]. A brief dietary interview was performed to ensure that all included subjects consumed adequate protein and energy.

Body composition including lean body mass (LBM) was measured by a DXA scan (Model DPX-IQ, Lunar Corp., Madison, WI, USA) at medium speed (24 mSv). Appendicular lean soft tissue (ALST) was calculated as lean soft tissue in arms and legs [41]. Skeletal muscle index (SMI) was calculated as ALST/height^2^ (H^2^). Furthermore fat-free mass/height^2^ (FFM/H^2^) was calculated [42] (see Table 1b).

**Table 1:** Baseline characteristics

	CON (n = 13)			RA (n = 13)			
A. Subject characteristics	Mean	SD		Mean	SD		*t* test
Age (years)	57	15		56	14		0.15
Height (cm)	169	5		167	4		0.32
Body mass index (BMI) (kg/m^2^)	25	5		25	4		0.85
Blood pressure (mmHg)	134/81	18/8		134/80	19/10		0.94
Waist/hip ratio	0.88	0.08		0.92	0.06		0.07
Resting heart rate (HR) (bpm)	70	12		65	10		0.11
B. Body composition	Mean	SEM		Mean	SEM		*t* test
Region % fat (fat %)	31.0	2.9		31.3	3.0		0.90
Lean body mass (LBM, kg)	45,2	2.1		44.5	1.3		0.65
Appendicular lean soft tissue (ALST, kg)	19,5	1.1		19.0	0.6		0.60
Skeletal muscle index (SMI, kg/m^2^)	6,8	0.3		6.8	0.2		0.54
Fat-free mass/height^2^ (FFM/H^2^) (kg/m^2^)	15.8	0.6		16.0	0.4		0.34
C. Strength and exercise	Mean	SEM	n	Mean	SEM	n	*t* test
1 repetition maximum (RM) exercised leg (kg)	38.4	3.0	13	37.6	2.5	13	0.73
Total kgs lifted	252	23	13	262	16	13	0.62
1 RM right leg (kg)	38.8	3.0	13	40.9	2.4	13	0.57
Maximal voluntary contraction (MVC) right leg (Nm)	176.1	10.2	11	179.9	16.6	8	0.71
Physical activity							
Physical Activity Scale (metabolic equivalent of tasks (METs)/24 hrs)	44.4	1.7	13	39.5	1.2	13	0.026

*CON* control subjects, *RA* rheumatoid arthritis patients

One repetition maximum (1 RM) was measured in a knee extension device (Technogym, Superexecutive Line, Gambottola, Italy) at range of motion 20°–100° (0° corresponds to full leg extension) and following individual adjustment and a brief warm-up consisting of low loads.

Maximal voluntary contraction (MVC) in isometric knee extension was determined for each leg at 70° of knee flexion using the Good Strength device (Version 3.14 Bluetooth; Metitur Ltd, Jyväskylä, Finland) after a 5-min warm-up on a stationary bike. The subjects were seated and fastened in a rigid chair with hips and knees flexed. A leg cuff, which was connected to a strain gauge through a rigid steel rod, was mounted on the leg just above the medial malleolus (Table 1c for strength data). Strength is expressed as moment in Nm, that is, corrected for lever arm length. The recorded moment was corrected for the effect of gravitational pull on the lower leg and foot by calibration before each measurement.

Resting blood samples were obtained by venipuncture for direct analysis of inflammatory cells and blood lipid profile at the Clinical Biochemistry Department, Bispebjerg Hospital, Copenhagen, using standard laboratory procedures. Furthermore, ethylenediaminetetraacetic acid (EDTA) plasma was stored at −80 °C pending analyses as described below.

### Experimental protocol

**Fig. 1:**
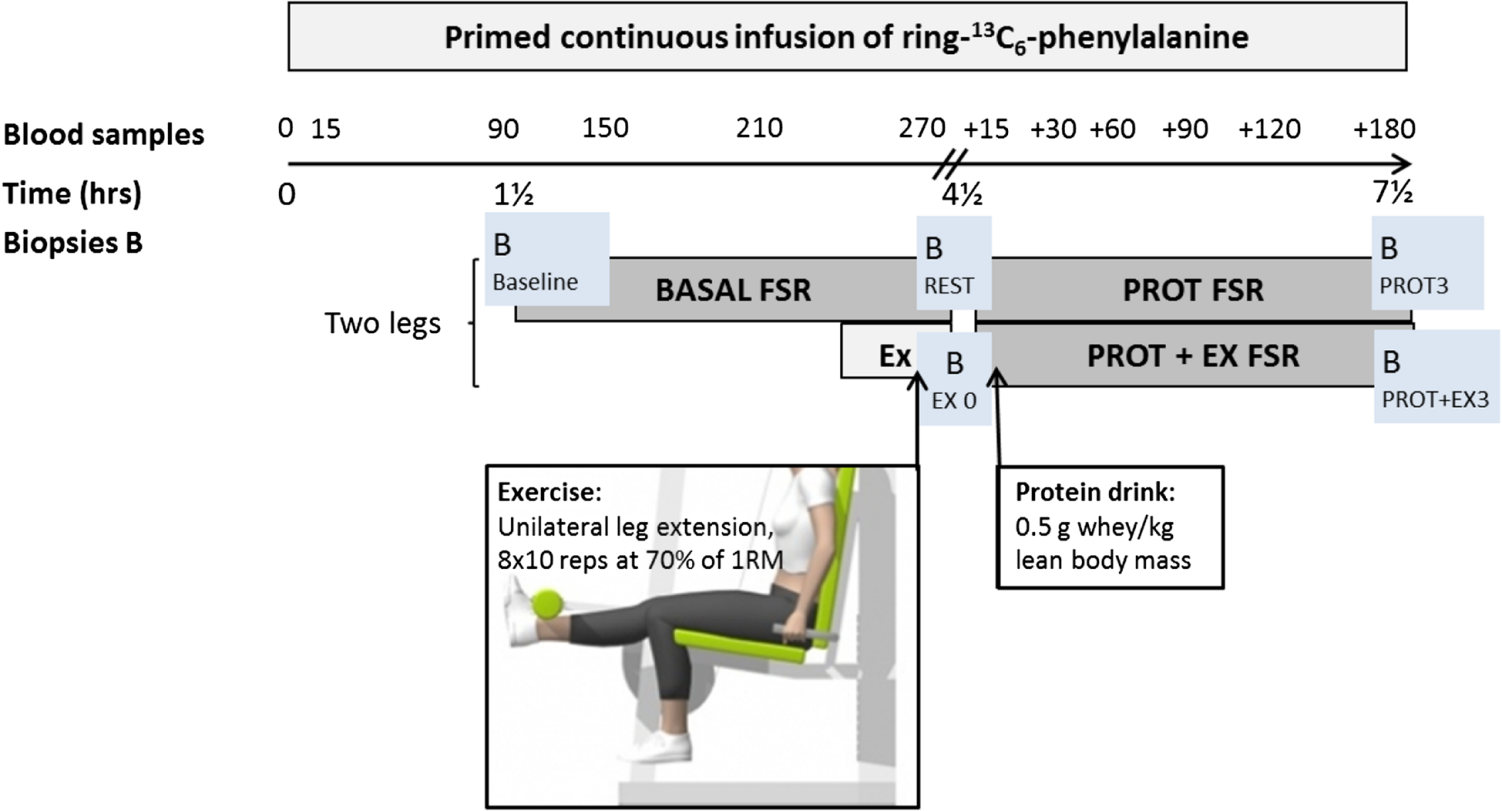
Study design. The stable isotope labeled amino acid ^13^C_6_-phenylalanine was infused throughout the study period, and fractional synthesis rate (FSR) of muscle protein (vastus lateralis) was measured in the resting, fasted (BASAL) state and after intake of whey protein alone (PROT) and in combination with unilateral resistance exercise (EX) (PROT + EX). *Upper line of grey boxes* represents one leg, *lower line* the contralateral leg. Muscle biopsy time points are marked with *B*

On the experimental day, subjects arrived fasted in the morning by taxi to the Institute of Sports Medicine, Bispebjerg Hospital, Copenhagen, Denmark, where they were placed supine and remained rested. A catheter was inserted into an antecubital vein of each arm; one used for tracer infusion and one used for collection of blood samples throughout the study. The trial design and sampling protocol is shown in Fig. 1. After obtaining a background blood sample, the ring-^13^C_6_-phenylalanine tracer (sterile and pyrogen-free; Cambridge Isotopes Laboratories, Andover, MA, USA) was administered as a primed (8 umol/kg LBM), continuous (8 umol/kg LBM/hr) infusion. The tracer, which was mixed in sterile saline and sterilized through a 0.2-μm sterile disposable filter (Minisart, Sartorius Stedium Biotech GmbH, Goettingen, Germany), was infused throughout the experimental day (total infusion time 8 hrs). After 1½ hrs of tracer infusion, the first muscle biopsy was obtained from the resting leg (B; baseline). At 4 hrs the subjects moved to the exercise equipment and (after one set of warm-up knee extensions consisting of eight repetitions at 35 % of 1 RM) performed one-legged knee extension exercises consisting of ten sets of eight repetitions at 70 % of 1RM separated by a 1-min break where subjects remained seated in the knee-extensor device. Subjects were randomized to perform the exercise with their dominant or non-dominant leg. The exercise session was completed in approximately 30 min and was supervised by the experiment leader. The contralateral leg remained rested. Immediately after cessation of the exercise session (4½ hrs of tracer infusion) biopsies were obtained from both legs, at least 4 cm away from the previous biopsy. Immediately hereafter a protein drink consisting of 0.5 g intact whey protein isolate (Lacprodan-9224, Arla Foods Ingredients, Viby, Denmark)/kg LBM (12.5 % enriched with ring-^13^C_6_-phenylalanine) dissolved in 190 ml water was consumed (total amount in RA and CON groups: 25.3 (0.7) and 25.7 (1.2) g (mean (SEM)), respectively). Three hours later bilateral biopsies were obtained, at least 4 cm away from any previous biopsies. The order of biopsies along one leg, and whether the exercised or rested leg was biopsied first was randomized among RA patients whereas identical procedures were followed in the matched CON subject.

### Blood samples and analyses

Venous blood samples were drawn into EDTA tubes and cooled on ice for 10 min, followed by centrifugation (10 min at 3060 g at 4 °C), and the plasma phase was stored at −80 °C until analysis.

Plasma levels of the inflammatory markers C-reactive protein (CRP), interleukin (IL)-6 and TNF-α were measured using enzyme-linked immunosorbent assay (ELISA) in blood samples obtained on the pretest day and the basal blood sample from the experimental day. The mean value of these is presented. ELISA kits (CRP DuoSet DY1707, IL-6 Quantikine HS ELISA Kit HS600B and TNF-α HSTA00D) were from R&D Systems (Minneapolis, MN, USA) and procedures have been described previously [43]. Inflammatory cell profile and blood lipid profile (triglycerides, total cholesterol, high-density lipoprotein (HDL) and low-density lipoprotein (LDL) cholesterol) in basal blood samples was measured at the Clinical Biochemistry Department, Bispebjerg Hospital, Copenhagen, as previously described [43].

During the experimental day blood samples (see Fig. 1) were obtained at time points 0, 15, 90, 150, 210 and 270 min after commencement of isotope infusion and at time points +15, +30, +60, +90, +120 and +180 min after consumption of the protein drink, for determination of ring-^13^C_6_-phenylalanine enrichment and plasma glucose. Blood glucose was measured immediately using an Accu-Chek, Inform II (Roche Diagnostics, Basel, Switzerland).

### Muscle biopsies

As shown in Fig. 1, a total of five biopsies (*B*) were obtained; baseline, rest, exercise (EX) 0, PROT 3 and PROT + EX 3 (at time points corresponding to 1½, 4½ and 7½ hrs of isotope infusion) providing three intervals for fractional protein synthesis rate (FSR) calculations; BASAL FSR (rest), PROT FSR (0–3 hrs after protein drink in rested leg), PROT + EX FSR (0–3 hrs after protein drink in exercised leg). The same five muscle biopsies were used for gene expression analyses by real-time reverse transcriptase polymerase chain reaction (RT-PCR) as described below. The muscle biopsies were obtained under local anesthetic (1 % lidocaine), through separate incisions at least 4 cm apart, in order to separate them as much as possible while still obtaining biopsies from reasonably similar areas of muscle, this has previously been shown not to affect FSR measurements [44]. The percutaneous needle biopsy technique with a 5-mm biopsy needle [45] and manual suction was used. The biopsy was freed from visible fat and connective tissue, and one piece immediately snap frozen (for later gene expression analyses) and another piece of approximately 30 mg (range 15.1–57.5) for stable isotope enrichments was wiped clean from blood in ice-cold saline, weighed, and snap frozen. Muscle biopsies were stored at −80 °C until analyses.

### Stable isotope analyses

#### Protein fractionation

Raw skeletal muscle specimens were homogenized (Fast-prep, 120A-230; Thermo Savant, Holbrook, NY, USA) for 4 × 15 sec in 1 ml homogenization buffer (0.02 M Tris, pH 7.4, 0.15 M NaCl, 2 mM EDTA, 0.5 % Triton-X 100, 0.25 M sucrose) left overnight at 5 °C, then homogenized once again at day 2 and left at 4 °C for 1 hr before centrifugation (20 min, 1600 g, 4 °C). The supernatant was discarded and 1.5 mL of high-salt buffer (0.7 M KCl and 0.1 M pyrophosphate) was added to the pellet, which was vortexed for 30 sec and left at 4 °C overnight. After a spin (20 min, 1600 g, 4 °C), the supernatant (myofibrillar protein fraction) was transferred to new vials and the pellet (connective tissue fraction) washed once more with high-salt buffer and left for 2 hrs and centrifuged (20 min, 1600 g, 4 °C) again from which the supernatant was discarded. The myofibrillar proteins in the supernatant were precipitated by adding 3.45 mL ice-cold 99 % ethanol and left at 4 °C for 30 min. After spinning (20 min, 1600 g, 4 °C) the supernatant was discarded. Both myofibrillar and connective tissue protein pellets were added to 1 mL of 6 M HCl and left at 110 °C overnight to hydrolyze proteins. The analysis of protein-bound tracer abundances were carried out on the GC-C-IRMS equipment (Finnigan Delta Plus, Bremen, Germany) as described in more detail elsewhere [46].

#### Precursor enrichment

Plasma-free amino acids were purified on resin columns (AG 50 W-X8 resin; Bio-Rad Laboratories, Hercules, CA, USA). After being washed, eluted and dried down under a stream of nitrogen, the purified amino acids were derivatized using *N*-methyl-*N*-(*tert*-butyldimethylsilyl)trifluoroacetamide + 1 % *tert*-butyl-dimethylchlorosilane (Regis Technologies, Morton Grove, IL, USA) mixed 1:1 with acetonitrile. The MTBSTFA-derivative of phenylalanine was separated on a CP-Sil 8 CB capillary column (30 m, 0.32 mm ID; coating, 0.25 μm) (ChromPack; Varian, Palo Alto, CA, USA) and the isotope ratios were analyzed on a triple-stage quadrupole mass spectrometer (TSQ Quantum; Thermo Scientific, San Jose, CA, USA) operated in electron ionization mode. Chromatogram integration was carried out in MassRatio 2.72 (FBJ Engineering, Frederiksværk, Denmark) and the tracer-to-tracee ratio (TTR) was calculated by subtracting the isotope ratio of a background sample from all the enriched samples.

#### Fractional synthesis rate calculations

The ring-^13^C_6_-phenylalanine enrichment of the myofibrillar and connective tissue muscle protein fractions measured by GC-C-IRMS (Hewlett Packard 5890-Finnigan gas chromatography-combustion III-Finnigan Delta^plus^ isotope ratio mass spectrometry; Thermo Finnigan MAT, Bremen, Germany) were used to calculate the fractional synthesis rate (FSR) in percent per hour. Calculations are based on the incorporation rate of ring ^13^C_6_-phenylalanine into muscle proteins using a standard precursor–product model as follows:

$$ \mathrm{F}\mathrm{S}\mathrm{R}\;\left(\%/ hr\right)=\frac{\Delta \mathrm{Eproduct}\times 100}{\mathrm{Eprecursor}\times \Delta \mathrm{t}} $$

where ΔEproduct is the change in tracer enrichment of protein-bound ring ^13^C_6_-phenylalanine in two biopsies from the same leg taken with a time interval of Δt. Eprecursor is the mean precursor ^13^C_6_-phenylalanine enrichment during that time interval. Here we used venous plasma tracer enrichments as a surrogate estimate of the precursor enrichment.

Whole-body rate of appearance (Ra) of ^13^C_6_-phenylalanine (an estimate of whole-body protein breakdown rate) was calculated as:

$$ \mathrm{R}\mathrm{a}=\frac{\mathrm{Infusion}\kern0.24em \mathrm{rate}}{\mathrm{E}\;\mathrm{plateau}} $$

Where Eplateau was the weighted average of venous plasma enrichment throughout the basal or protein (PROT) + exercise (EX) periods or the two combined.

### RNA extraction

RNA was extracted as described in [47]. Essentially, approximately 15 mg of frozen muscle tissue from each biopsy was homogenized in TRI Reagent (Molecular Research Center, Cincinnati, OH, USA), using 1-bromo-3-chloropropane for phase separation and isopropanol to precipitate RNA. The RNA pellet was washed in ethanol and dissolved in RNase-free water. RNA concentrations were determined by spectroscopy at 260 nm. Quality of the RNA was checked by gel electrophoresis and spectrophotometer ratios at 260/240 nm (acceptable range 1.2–1.6 at pH 8) and 260/280 nm (1.8–2.0 at pH 7.5–8.0).

### Real-time RT PCR analysis

Expression of a total of 26 different genes was measured (see Table 2 for full list) belonging to the following groups; satellite cell regulators and inflammation, heat shock proteins, myogenic regulatory factors, atrogenes and cytokines and their receptors.

**Table 2:** Primers used for real-time RT-PCR

Target	Sense primer	Antisense primer
RPLP0	GGAAACTCTGCATTCTCGCTTCCT	CCAGGACTCGTTTGTACCCGTTG
GAPDH	CCTCCTGCACCACCAACTGCTT	GAGGGGCCATCCACAGTCTTCT
IGF-IEa	GACATGCCCAAGACCCAGAAGGA	CGGTGGCATGTCACTCTTCACTC
IGF-IEc	GCCCCCATCTACCAACAAGAACAC	CGGTGGCATGTCACTCTTCACTC
HGF	TGAAATATGTGCTGGGGCTGAAA	ACAAACAAGTGGGCCACCATAATCC
Cmet	AACCCGAATACTGCCCAGACCC	TGATATCCGGGACACCAGTTCAG
MCP1	GCCCTTCTGTGCCTGCTGCT	GCAGGTGACTGGGGCATTGATT
COX1	GGTTTGGCATGAAACCCTACACCT	CCTCCAACTCTGCTGCCATCT
COX2	TGGAACATGGAATTACCCAGTTTGTTG	TGTGATACTTTCTGTACTGCGGGTGG
HSP70	GTGGCTGGACGCCAACACCTT	TTACACACCTGCTCCAGCTCCTTC
HSP27	GCTGACGGTCAAGACCAAGGATG	TGAAGCACCGGGAGATGTAGCC
αB-crystallin	GTGTTGGGAGATGTGATTGAGGTG	CTGGGATCCGGTATTTCCTGTGG
Myogenin	CTGCAGTCCAGAGTGGGGCAGT	CTGTAGGGTCAGCCGTGAGCAG
Myf6	GGGCTCGTGATAACGGCTAAGGA	TGTCCACGATGGAAGAAAGGCA
MyoD	ACGAAGGCGCCTACTACAACGA	GACACCGCCGCACTCTTCCC
Myostatin	TGCTGTAACCTTCCCAGGACCA	GCTCATCACAGTCAAGACCAAAATCC
Atrogin1	TGTTACCCAAGGAAAGAGCAGTATGGA	ACGGAGCAGCTCTCTGGGTTATTG
MuRF1	TGGGGGAGCCACCTTCCTCT	ATGTTCTCAAAGCCCTGCTCTGTCT
TNFα	TTCCCCAGGGACCTCTCTCTAATC	GAGGGTTTGCTACAACATGGGCTAC
TNFRI	GGGAGGACAGCGCCCACAAG	CACGAATTCCTTCCAGCGCAAC
TNFRII	CCACTCGGAACCAGCCACAG	CCATGGCCACCAGGGGAAGA
IL-1β	TGCGTGTTGAAAGATGATAAGCCCA	CAAATCGCTTTTCCATCTTCTTCTTTG
IL-1R	GGAAGGGATGACTACGTTGGGGA	CCAGCCAGCTGAAGCCTGATGTT
IL-6	GAGGCACTGGCAGAAAACAACC	CCTCAAACTCCAAAAGACCAGTGATG
IL-6R	CCAGGAGGAGTTCGGGCAAG	GGGGTGGACACCTCGTTCTCA

*Abbreviations: RT-PCR* real-time reverse transcriptase-polymerase chain reaction, *RPLP0* large ribosomal protein P0, *GAPDH* glyceraldehyde-3-phosphate dehydrogenase, *IGF* insulin-like growth factor, *HGF* hepatocyte growth factor, *MCP1* monocyte chemoattractant protein = CCL2, *COX* cyclooxygenase, *HSP* heat shock protein, *MuRF1* muscle ring finger protein 1, *TNF* tumor necrosis factor, *IL* interleukin, *R* receptor

The HGF primers are specific for the two largest splice variants including the SP domain

Total RNA (500 ng from each muscle sample) was converted into cDNA in 20 ul using the OmniScript reverse transcriptase (Qiagen, Valencia, CA, USA) according to the manufacturer’s protocol.

For each target mRNA, 0.25 ul cDNA was amplified in a 25-ul SYBR Green PCR reaction containing 1 × Quantitect SYBR Green Master Mix (Qiagen) and 100 nM of each primer (Table 2). The amplification was monitored real time using the MX3005P real-time PCR machine (Stratagene, Santa Clara, CA, USA). The threshold cycle (Ct) values were related to standard curves made with PCR products to determine the relative difference between the unknown samples, accounting for the PCR efficiency. The specificity of the PCR products was confirmed by dissociation curve analysis after amplification. All mRNA data were normalized to ribosomal protein, large, P0 (RPLP0). For normalization control, glyceraldehyde-3-phosphate dehydrogenase (GAPDH), see Additional file 1. Baseline data (1.5 hrs rest, relative to mean CON) are shown in Additional file 2. mRNA expression data are presented as fold changes relative to individual baseline values.

### Statistical analysis

Results are reported as mean (SE) unless otherwise stated. Protein synthesis and gene expression data were analyzed by two-way repeated-measures (RM) ANOVA (SigmaPlot 12.3, Systat Software Inc, San Jose, CA, USA) and Student-Newman-Keuls (SNK) post hoc test with correction for multiple comparisons. Significant effects of group (CON vs. RA) or biopsy and interaction (group × biopsy) are shown on graphs at a significance level of *p* < 0.05. *P* values ≤0.1 are also shown on graphs, for trends. All mRNA data were log-transformed for statistical analyses and shown as geometric mean ± back-transformed SE. For some mRNA targets, the pattern of missing data led to exclusion of subject pairs from the statistical analyses, resulting in exclusion of the following number of subject pairs; IL1β = 4; IGF-IEc = 1; IL1R = 1; cmet = 1. All other data (with only a single data point per subject) were compared by a paired two-tailed *t* test (Prism 6.02 for Windows, GraphPad Software Inc, La Jolla, CA, USA). Plasma ELISA data were log-transformed for statistical analyses.

## Results and discussion

### Baseline characteristics

**Fig. 2:**
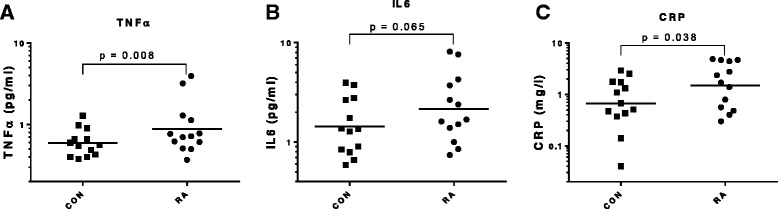
Systemic inflammatory markers. Plasma levels of the inflammatory markers tumor necrosis factor α (TNF-α), interleukin 6 (IL-6) and C-reactive protein (CRP) measured in controls (CON) and RA patients (RA). Individual data (mean of two sampling days) were log-transformed for statistical analyses and data are shown on log scales with line at geometric means. Significance level of paired comparisons is given on graphs

As shown in Fig. 2, CRP was higher in the RA patients (2.25 (0.50) mg/l) than in CON subjects (1.07 (0.25) mg/l; *p* = 0.038), TNF-α was higher in RA (1.18 (0.30) pg/ml) than CON (0.64 (0.07) pg/ml; *p* = 0.008) and IL-6 tended to be higher in RA (RA 2.89(0.68) pg/ml; CON 1.74(0.32) pg/ml; *p* = 0.065). Although going in the same direction, these differences in inflammatory markers between RA patients and healthy CON individuals were not as pronounced as previously reported in a majority of studies [32, 35, 37, 38, 48, 49], but were similar to the moderate levels observed by Crowson et al. [50]. Most likely, the limited elevation of systemic inflammatory markers emphasizes the very well-functioning state of the RA patients participating in this study.

**Fig. 3:**
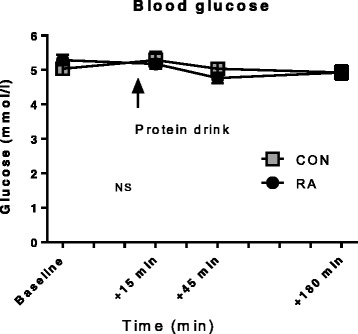
Blood glucose levels. Level of blood glucose throughout the experimental day. Subjects arrived fasted in the morning, and ingested only the protein drink (0.5 g whey/kg lean body mass) as marked by the *arrow*. Mean ± SE, n = 6–12

The overview of subject characteristics in Table 1 reveals many similarities between RA and CON groups. However, waist/hip ratio (Table 1a) tended to be higher in RA than CON (*p* = 0.07). Among RA patients, six were smokers (5–20 cigarettes/day, mean 10) and among controls four were smokers (1–17 cigarettes/day, mean 10). Use of medication in the two groups is shown in Table 3. These records show that all RA patients use medication of some type, mostly DMARDs like methotrexate (all 13 patients) and salazopyrin (5/13 patients). Pain relief medication was frequently used by RA patients, mostly paracetamol (7/13 patients) and nonsteroidal anti-inflammatory drugs (NSAIDs) (3/13) whereas only a single CON participant reported use of each of these drugs. Most RA patients had some comorbidity (see Additional file 3 for complete list), except for two patients. No subjects from the CON group used any rheumatic drugs and had only a minor intake of painkillers. Seven CON subjects were completely free from comorbidities. These differences in comorbidities and medication could be confounding the systemic inflammation data (Fig. 2), since some comorbidities may contribute to an elevation of inflammatory markers while antirheumatic medication is likely to reduce these markers. Body composition measures (Table 1b) were not different between RA and CON, likewise, no differences in knee extensor muscle strength (Table 1c) measured as one repetition maximum (1 RM) and maximal voluntary contraction (MVC) were observed between groups. The total number of kg lifted during the experimental acute exercise session was not different between groups (Table 1c). When estimated by PAS, physical activity level turned out higher in CON than in RA (*p* = 0.026). The similarities in body composition between RA patients and CON were somewhat surprising and in contrast to previous reports of reduced muscle mass [2, 16, 42] and increased fat accumulation in RA patients [51, 52]. Furthermore, the similar muscle strength between RA and CON indicate that the patients were well-functioning in comparison to those participating in previous studies [16, 17, 38, 53, 54]. Data on blood lipid profile and circulating inflammatory cells is given in Table 4. Although metabolic changes are usually reported at all stages of RA disease [19], we detected neither differences between RA and CON in blood lipid profile, blood pressure, metabolic syndrome biomarkers, fasting glucose, nor in the circulating inflammatory cell profile for all of which changes have previously been reported in RA patients [19, 48, 50]. Again, this reflects the clinically well-controlled condition of the participating patients. Throughout the experimental day, blood glucose level was not different between groups and was stable around 5 mmol/l (Fig. 3 and Table 4b).

**Table 3:** Medication

	Medication	
	CON	RA
Methotrexate	0	13
Salazopyrin	0	5
Paracetamol	1	7
NSAID	1	3
Acetylsalicylic acid	2	0
Anti-asthmatics	0	4
Folic acid	0	8
Statins	1	1
ACE inhibitors	0	3
Hormone therapy	0	1
Pain relief (tramadol)	0	1
No medication	9	0

*Abbreviations: CON* control subjects, *RA* rheumatoid arthritis patients, *NSAID* nonsteroidal anti-inflammatory drug, *ACE* angiotensin-converting enzyme

**Table 4:** Inflammaroty cells & Blood lipid profile

	CON (n = 13)		RA (n = 13)		
A. Inflammatory cell profile	Mean	SEM	Mean	SEM	*t* test
Leucocytes (total, x10^9^/l)	7.3	0.4	6.7	0.7	0.53
Neutrohils (x10^9^/l)	4.7	0.3	4.1	0.6	0.44
Lymphocytes (x10^9^/l)	1.81	0.13	1.82	0.19	0.96
Monocytes (x10^9^/l)	0.57	0.05	0.55	0.05	0.77
B. Blood lipid profile					
Triglycerides (mmol/l)	1.2	0.1	1.4	0.2	0.41
Total cholesterol (mmol/l)	5.3	0.3	5.5	0.3	0.75
HDL cholesterol (mmol/l)	1.7	0.1	1.6	0.1	0.44
LDL cholesterol (mmol/l)	3.1	0.2	3.4	0.3	0.28
Fasting glucose (mmol/l)	5.0	0.2	5.3	0.2	0.66

*Abbreviations: CON* control subjects, *RA* rheumatoid arthritis patients, HDL, high-density lipoprotein, LDL, low-density lipoprotein

### Muscle protein synthesis

**Fig. 4:**
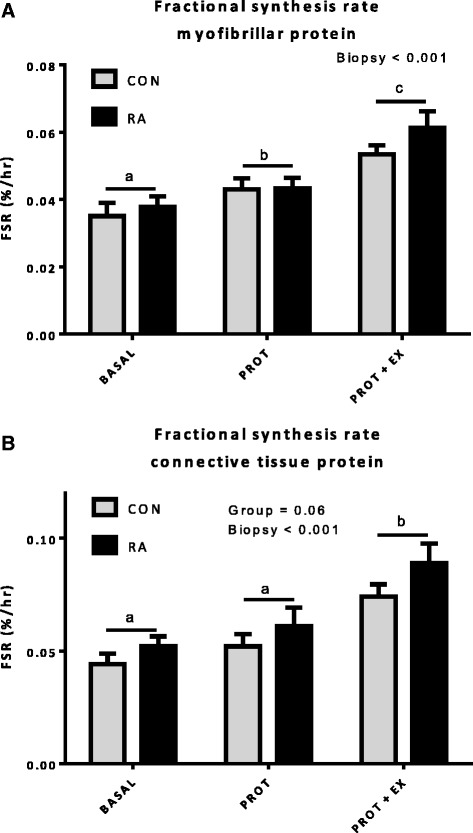
Fractional synthetis rate (FSR) of muscle myofibrillar and connective tissue protein. FSR of muscle myofibrillar (**a**) and connective tissue (**b**) protein given in %/hr in control (CON) and rheumatoid arthritis (RA) patient groups, measured in the resting, fasted (BASAL) state and after intake of whey protein alone (PROT) and in combination with unilateral resistance exercise (EX) (PROT + EX). *Black bars* denote rheumatoid arthritis patients (RA, n = 13) and *grey bars* healthy controls (CON, n = 12–13). Letters *a*, *b* and *c* denote significant differences between sampling time points (two-way RM ANOVA)

**Fig. 5:**
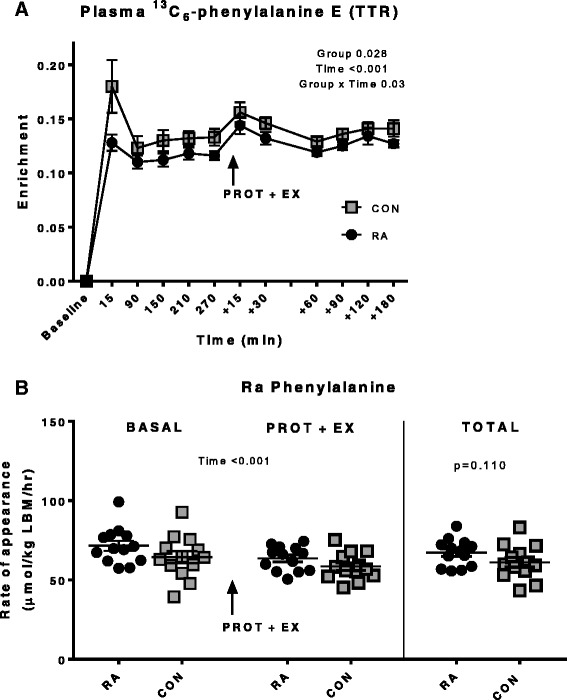
Enrichment and rate of appearance of ^13^C_6_-phenylalanine. **a** Plasma ^13^C_6_-phenylalanine (^13^C_6_-Phe) enrichment (tracer-to-tracee ratio (TTR)) during the entire infusion period. Mean ± SE, n = 13. **b** Rate of appearance (μmol/kg LBM/hr) of ^13^C_6_ Phe (Ra) during the basal period (BASAL, 3 hrs), after protein intake and exercise (PROT + EX, 3 hrs) and over the two periods combined (TOTAL, 6 hrs). Over the total period, Ra tended to be higher in rheumatoid arthritis patients (RA) vs. healthy controls (CON) (*p* = 0.110). Individual data are shown with line at mean ± SE, n = 13

Fractional synthesis rate (FSR) of muscle myofibrillar and connective tissue protein is shown in Fig. 4. The myofibrillar protein synthesis was enhanced in response to protein intake (*p* < 0.05) and was further increased when combined with heavy resistance exercise (*p* < 0.001). This response was similar in CON and RA groups. Connective tissue protein synthesis was increased after exercise combined with protein intake (*p* < 0.001), but not by protein intake alone (*p* > 0.1). Irrespective of state (fasting, protein fed alone or in combination with exercise), connective tissue FSR tended to be higher in RA than CON (*p* = 0.060). Plasma tracer enrichment (Fig. 5) was lower in RA vs. CON throughout the infusion period (*p* = 0.028) (ranged between 0.11 and 0.14 in RA and 0.12 and 0.18 in CON). Whole-body protein breakdown rate (rate of tracer amino acid appearance) was reduced following protein intake (PROT) and one-legged resistance exercise (EX) (BASAL = 64.4 (SE 3.8) and 71.5 (SE 3.2) and PROT + EX = 58.5 (SE 2.4) and 63.6 (SE 2.1) μmol/kg LBM/hr in CON and RA, respectively; time *p* < 0.001) and tended to be higher in RA than in CON as an average over the entire study period (TOTAL; *p* = 0.11). However, the whole-body assessment of protein turnover is neither protein nor tissue specific and we cannot say whether the tendency to a higher protein turnover rate in RA patients is a general phenomenon or may be related to a specific tissue (i.e., skeletal muscle) or protein type.

We showed a comparable basal muscle protein synthesis rate in RA and CON (Fig. 4a and b). Muscle protein turnover in patients with RA has to our knowledge been investigated in only one human study previously [55], showing that the resting, fasted FSR in RA patients not receiving steroid therapy was similar to osteoarthritis patients serving as controls. Further, the present study shows for the first time an anabolic response (elevated myofibrillar FSR) to acute whey protein feeding alone, which was amplified when combined with acute resistance exercise in patients with RA. Additionally, this response was not different from that observed in healthy control subjects matched for age, gender, BMI and physical activity. Apparently, in our RA patients the connective tissue fraction was less responsive to nutritional intervention than the myofibrillar fraction, which is in accordance with previous findings [56]. For further details of the anabolic response to acute exercise and protein feeding, protein expression and signaling analyses of targets of the Akt-mTOR signaling pathway would have been relevant, however, since we observed similar FSR responses in RA and CON, we chose to focus on transcriptional regulation of genes involved in other aspects of muscle adaptation as described in the following section.

### Muscle gene expression

**Fig. 6:**
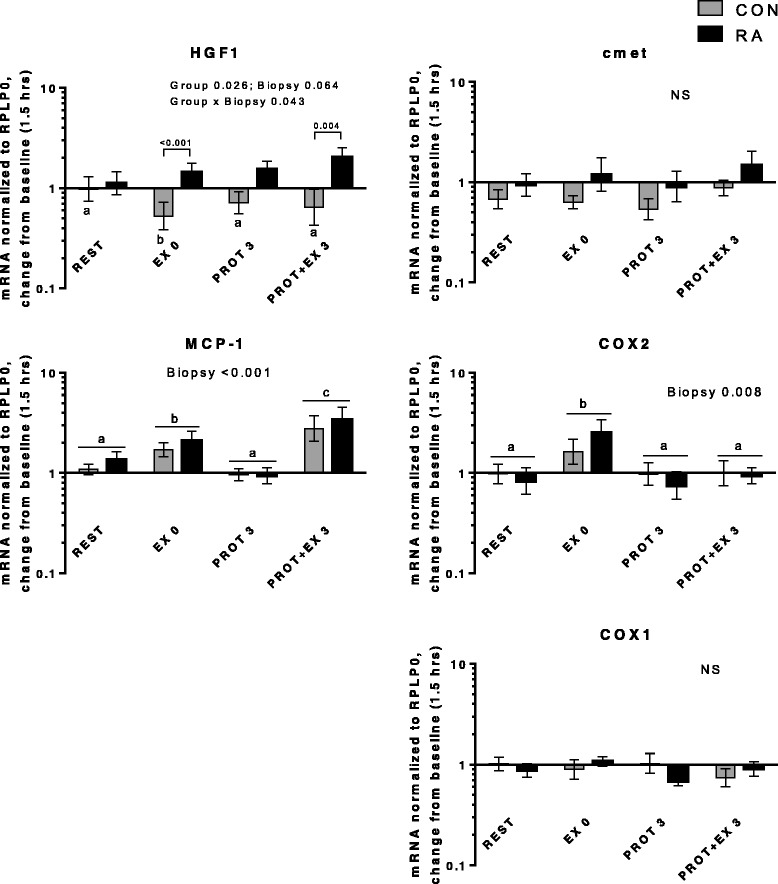
Satellite cell regulators and inflammation. mRNA expression relative to 1.5 hrs (baseline). For hepatocyte growth factor 1 (HGF1), letters *a-b* denote significant differences within controls (CON)

**Fig. 7:**
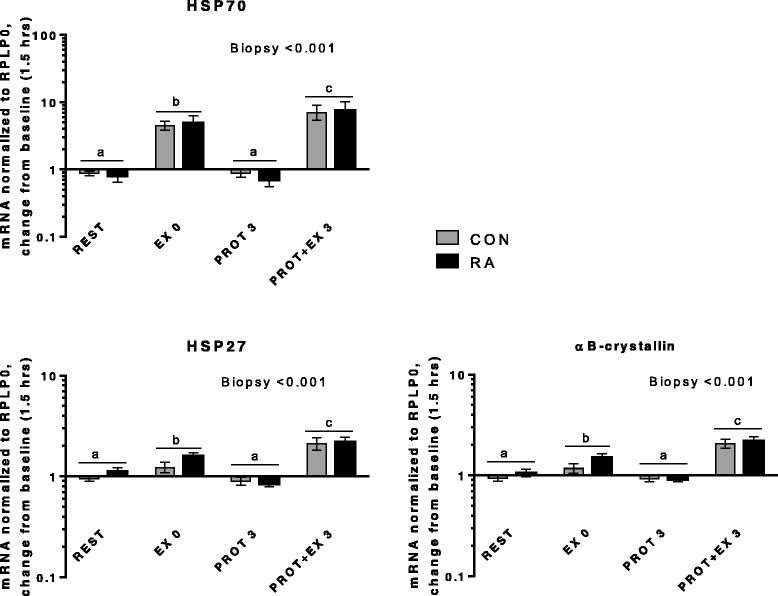
Heat shock proteins. mRNA expression relative to 1.5 hrs (baseline)

**Fig. 8:**
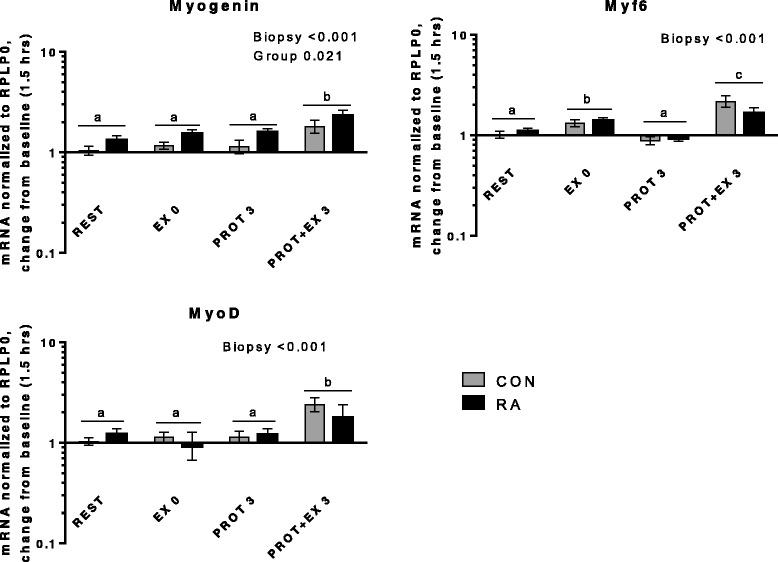
Myogenic regulatory factors. mRNA expression relative to 1.5 hrs (baseline). Myogenin expression at REST was significantly different from baseline

**Fig. 9:**
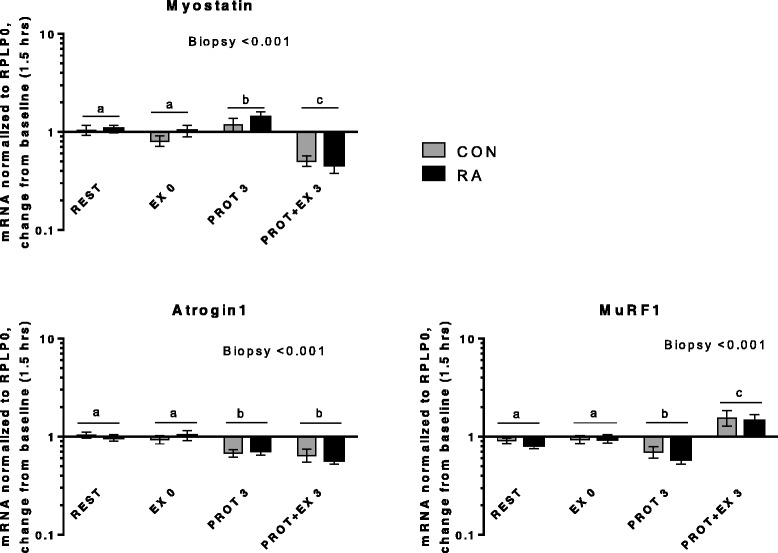
Atrogenes. mRNA expression relative to 1.5 hrs (baseline)

**Fig. 10:**
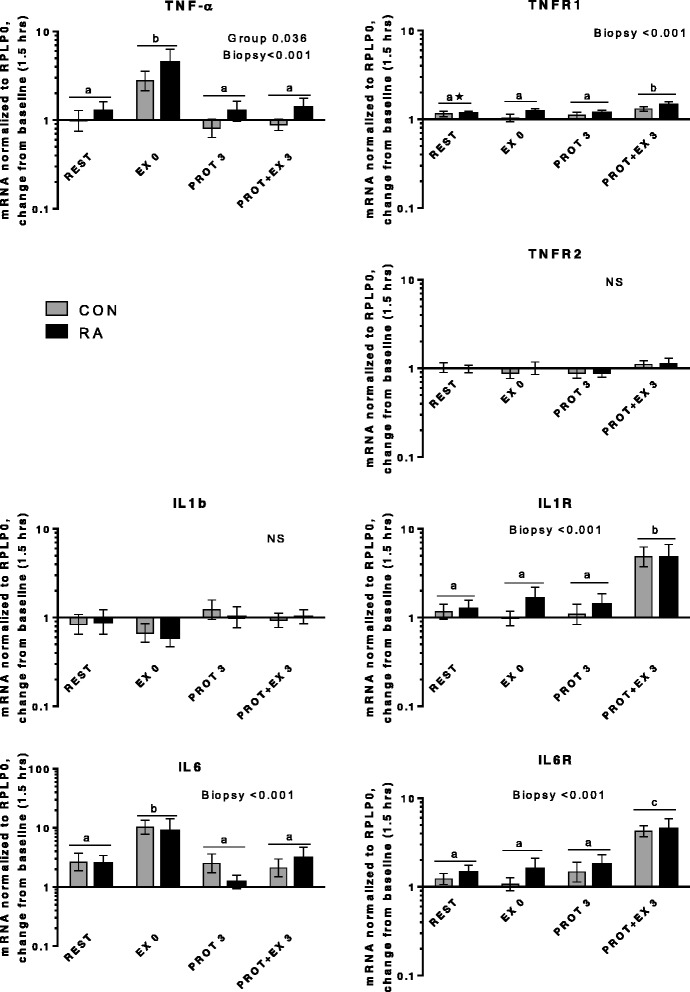
Cytokines and receptors. mRNA expression relative to 1.5 hrs (baseline). Tumor necrosis factor receptor 1 (TNFR1) and interleukin (IL)-6 expression at REST was significantly different from baseline

In the present study, expression of genes related to inflammatory signaling, myogenesis and muscle growth/atrophy as well as heat shock proteins responded similarly in RA and CON. No differences were observed in basal gene expression level between RA and CON (Additional file 2). Changes in mRNA expression from baseline is shown in Figs. 6, 7, 8, 9 and 10, divided into subgroups related to satellite cell (SC) regulators and inflammation (Fig. 6), heat shock proteins (Fig. 7), myogenic regulatory factors (Fig. 8), atrogenes (Fig. 9), as well as cytokines and receptors (Fig. 10). As shown in Fig. 6, HGF1 expression was overall higher in RA vs. CON (Group, *p* = 0.026), specifically HGF1 expression was higher in RA patients than CON at EX0 and PROT + EX3 (*p* < 0.001 and *p* = 0.004, respectively). The higher expression of HGF1 in RA than CON indicate an increased sensitivity toward signaling via this pathway in RA patients, which could be located to the skeletal muscle stem cells, SCs, since HGF1 signaling is involved in activation of SC [57, 58], although our gene expression analysis is not specific to the SCs. HGF1 activates SCs via binding to the cmet receptor [59].

However, no significant changes in gene expression of the HGF1 receptor, cmet, were observed, indicating that this is not the regulatory site for this pathway. Macrophage chemoattractant protein 1 (MCP-1, also known as CCL2) was induced by exercise (but not protein feeding), both acutely (EX 0, *p* = 0.02) and even more 3 hrs later (*p* < 0.001) in both groups combined. This indicates that it is involved in the adaptive response to resistance exercise. Potentially, it plays a role in crosstalk between inflammatory cells (macrophages) and SC, as indicated by its colocalization with these cells [60]. Cyclooxygenase 2 (COX2) expression was induced immediately after exercise (*p* = 0.011), in line with previous reports [61, 62], although at later time points. In contrast, we and others have previously reported COX2 induction only when exercise was combined with COX inhibition [47, 63]. Taken together, we observed some indications on involvement of HGF1, MCP-1 and COX2 in the adaptive response to exercise, however, differential regulation between RA and CON was only observed for HGF1.

In Fig. 7 mRNA expression of heat shock proteins (HSPs) is shown. All three HSPs (HSP70, HSP27 and αB-crystallin) were induced by exercise both immediately after (EX 0, *p* < 0.001) and 3 hrs later (PROT + EX 3, *p* < 0.001), but not by protein intake. The induction of HSPs a few minutes after exercise (EX0), suggests that the HSP response to unaccustomed exercise is even more acute than previously shown (as discussed in [64]) and that muscle of RA patients is equally responsive as in CON.

Myogenic regulatory factors (Fig. 8) were induced by exercise combined with protein intake but not by protein intake alone. Myogenin expression was higher in RA than CON (Group; *p* = 0.021), pointing at an increased responsiveness in RA patients, although this was not apparent for the other myogenic regulatory factors Myf6 and MyoD. Myf6 expression was increased both immediately after exercise (EX 0, *p* < 0.001) and 3 hrs later (PROT + EX 3, *p* < 0.001). Expression of MyoD was increased only 3 hrs after exercise (PROT + EX 3, *p* < 0.001). In general, myogenic regulatory factors were induced by exercise, and mainly after 3 hrs compared with immediately after, which is in line with previous observations [65, 66] and the response was not different between RA and CON. Nor did we observe a difference in resting gene expression between RA and CON (Additional file 2) in the current study. In muscle from a rat model of RA (adjuvant-induced arthritis) both protein and mRNA expression of MyoD and myogenin were increased at rest, however, this was not investigated in relation to exercise [24–26].

Expression of myostatin and the atrogenes Atrogin1 and MuRF1 is shown in Fig. 9. The negative regulator of muscle mass, myostatin, was downregulated 3 hrs after exercise + protein (PROT + EX 3, *p* < 0.001) and responds to exercise in an overall similar manner in RA patients and CON. Atrogin1 was downregulated 3 hrs after protein intake alone (PROT 3, *p* < 0.001) and in combination with exercise (PROT + EX 3, *p* < 0.001), whereas MuRF1 was downregulated 3 hrs after protein intake alone (PROT 3, *p* < 0.001), but upregulated 3 hrs after exercise combined with protein intake (PROT + EX3, *p* < 0.001). Also for the atrogenes, no impact of RA could be observed. In muscle of the rat model of RA, mRNA expression of MuRF1 and atrogin1 was markedly increased [27, 29, 30], however, this difference was not apparent in our human subjects. Similarly, COX2 expression was markedly increased in muscle of arthritic rats, which was not reproduced in the RA patients of the present study either (Fig. 6).

Figure 10 displays mRNA expression of selected cytokines and their receptors. TNF-α expression was higher in RA than in CON across all biopsy points (Group, *p* = 0.036) and was induced immediately after exercise (EX 0, *p* < 0.001); the former is in line with the increased mRNA expression of TNF-α found in gastrocnemius muscle of rats with adjuvant-induced arthritis [27, 29] indicating a more responsive TNF-α expression in muscle from RA patients. TNF-α is believed to be a central mediator of muscle wasting in rheumatoid arthritis by alteration of the balance between muscle protein synthesis and breakdown. Via inhibition of signaling from the insulin receptor [67] and IGF-1 receptor via JNK and IRS-1 [68], TNF-α can reduce peripheral insulin action and interfere with IGF-1 signaling, leading to a reduction of the anabolic responsiveness. Anti-TNF-α therapies have proven effective in RA although muscle mass is not necessarily reversed by anti-TNF-α treatment [32]. However, the anabolic response to a positive energy balance was improved by anti-TNF-α treatment, seen by a larger gain of fat-free mass compared with methotrexate treatment [35] and supporting a role for TNF-α in the regulation of muscle mass. Interestingly, the higher TNF-α expression in the present study did not result in such differences in muscle mass or acute anabolic response, leaving the significance of this differential TNF-α expression an open question.

TNFR1 expression was slightly increased 3 hrs after protein + exercise (PROT + EX 3, *p* < 0.001), whereas no changes were observed for TNFR2 or IL-1β. Induction of TNFR expression 3 hrs after exercise + protein has to our knowledge not been reported before, although higher levels of TNFR1 gene expression was recently reported in older (61 and 76 years) compared to younger subjects (40 years) [69], both at rest and 24 hrs after acute resistance exercise. Generally, regulation of TNF receptors in human muscle is not well understood. Both TNFR1 and 2 were expressed at high levels in the present study, while correlations were observed between TNFR1 and 2 expression (r = 0.57, *p* = 0.003, data not shown). Inflammatory signaling via IL-6, IL-6R and IL-1R was induced by exercise with an early upregulation of IL-6 immediately after exercise (EX 0, *p* < 0.001) and to a lesser extent in the resting leg (REST, *p* < 0.001) whereas the receptors were upregulated 3 hrs after exercise + protein (PROT + EX 3, *p* < 0.001). None of these responses were different between RA and CON, indicating a normal cytokine response to acute resistance exercise in RA patients.

At baseline no differences in mRNA expression between RA and CON were observed for any of the investigated target genes (Additional file 2), although in skeletal muscle of arthritic rats, marked changes in gene expression induced by the disease have been consistently reported.

Thus, our human data from RA patients do not confirm the upregulation of muscle regulatory, inflammatory and catabolic markers found in animal models of RA, which is in line with the overall healthy state and preserved anabolic response of RA patients in the present study.

In contrast to the present findings in RA patients, remarkable differences in gene expression between elderly and young muscle previously have been reported including an elevated expression of inflammatory genes [69, 70], atrogenes [71] and MRFs [65] in resting skeletal muscle. Within the same time frame as used in the present study, Atrogin1 is induced by exercise only in old muscle [71], and IL-6 induction and myostatin downregulation by resistance exercise are more pronounced in old compared to young muscle [70, 72]. Together these results from elderly muscle indicate increased muscle inflammation susceptibility [69] and an altered acute muscle adaptive response to exercise in elderly muscle, which could also contribute to the muscle deficits in RA patients. However, apart from a more pronounced induction of TNF-α, HGF1 and myogenin in RA vs. CON, this was not the case in the present study. Previously, knowledge about regulation of muscle gene expression in RA has relied only on animal studies, but from the current study we can now add human data.

Taken together, our gene expression data indicate that specific targets involved in muscle and SC regulation (HGF1, myogenin and TNF-α) are induced to a larger extent in RA patients than in healthy CON subjects, however, the majority of genes investigated showed similar responses in RA vs. CON indicating that skeletal muscle tissue of RA patients responds equally well to an acute exercise stimulus compared to healthy CON subjects.

### Limitations

Keeping in mind that results may not apply for RA patients in general, the present study indicate that skeletal muscle of RA patients does not differ markedly from healthy control muscle and that they respond to protein intake alone and in combination with exercise in a similar way. The patients participating in the present study were a selected group of well-functioning RA patients, and thus no changes in either muscle strength or muscle mass were detected, which reduces the external validity of the study and leaves the question open of how RA patients with highly elevated systemic inflammatory levels and/or cachexia are characterized with respect to molecular (signaling) regulators of muscle mass and muscle protein turnover in response to the same interventions.

## Conclusions

In conclusion, muscle protein synthesis and transcriptional regulation can be stimulated with both protein intake and physical exercise in patients with RA to a similar degree as in healthy individuals. These findings show that characteristics inherent of RA disease do not affect the muscle protein synthesis and gene expression response to acute exercise and protein intake, when factors like BMI, age and activity level are controlled by carefully matching each patient with a corresponding healthy control subject.

## Additional files

Additional file 1:
**Normalization control.** GAPDH gene expression data normalized to RPLP0 and individual baseline values, log-transformed for statistical analyses and shown on a logarithmic scale as geometric mean ± SEM. *Black bars* denote rheumatoid arthritis patients (RA, n = 13) and *grey bars* healthy controls (CON, n = 13). (PDF 173 kb) Additional file 2:
**mRNA expression at baseline.** Baseline expression of all targets is expressed relative to mean healthy CON at baseline. Gene expression data were normalized to RPLP0, log-transformed for statistical analyses and shown on a logarithmic scale as geometric mean ± SEM. *Black bars* denote rheumatoid arthritis patients (RA, n = 13) and *grey bars* healthy controls (CON, n = 13). No significant differences were observed between groups. (PDF 177 kb) Additional file 3:
**Comorbidities.** Table of reported comorbidities in rheumatoid arthritis patients (RA) and healthy controls (CON). The number of subjects reporting each comorbidity is given. (PDF 179 kb)

## Abbreviations

ACR: American College of Rheumatology
ALST: appendicular lean soft tissue
B: baseline
BMI: body mass index
CON: control
COPD: chronic obstructive pulmonary disease
COX: cyclooxygenase
CRP: C-reactive protein
DAS28: Disease Activity Score in 28 joints
DMARD: disease-modifying antirheumatic drug
DXA: dual-energy X-ray absorption
E: enrichment
EDTA: ethylenediaminetetraacetic acid
ELISA: enzyme-linked immunosorbent assay
EX: exercise
FFM: fat-free mass
FSR: fractional synthesis rate
GAPDH: glyceraldehyde-3-phosphate dehydrogenase
H: height
HDL: high-density lipoprotein
HGF: hepatocyte growth factor
HSP: heat shock protein
IGF: insulin-like growth factor
IL: interleukin
LBM: lean body mass
LDL: low-density lipoprotein
MCP1: monocyte chemoattractant protein = CCL2
MET: metabolic equivalent of task
MuRF1: muscle ring finger protein 1
MVC: maximal voluntary contraction
NSAIDs: nonsteroidal anti-inflammatory drugs
PAS: Physical Activity Scale
PROT: protein
R: receptor
Ra: rate of appearance
RA: rheumatoid arthritis
RM: repetition maximum
RPLP0: large ribosomal protein P0
RT-PCR: reverse transcriptase-polymerase chain reaction
SCs: satellite cells
SMI: skeletal muscle index
TNF: tumor necrosis factor
TTR: tracer-to-tracee ratio
