# Supplementary material for: Preserved skeletal muscle protein anabolic response to acute exercise and protein intake in well-treated rheumatoid arthritis patients
Source: Arthritis Res Ther. 2015 Sep 25;17:271. doi: 10.1186/s13075-015-0758-3 (PMC4583143; doi:10.1186/s13075-015-0758-3)

Additional file 2. Normalisation control

GAPDH gene expression data normalized to RPLP0 and individual baseline values, log-transformed for statistical analyses and shown on a logarithmic scale as geometric mean ±SEM. Black bars denote rheumatoid arthritis patients (RA, n=13) and grey bars healthy controls (CON, n=13).

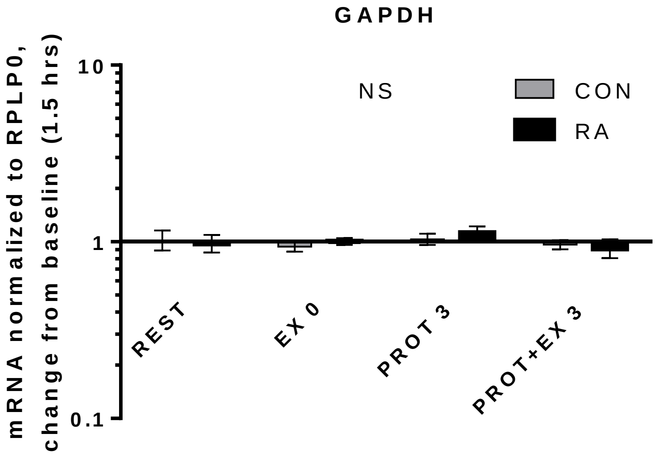

Supplement: Additional file 1: — Normalization control. GAPDH gene expression data normalized to RPLP0 and individual baseline values, log-transformed for statistical analyses and shown on a logarithmic scale as geometric mean ± SEM. Black bars denote rheumatoid arthritis patients (RA, n = 13) and grey bars healthy controls (CON, n = 13). (PDF 173 kb) [file 13075_2015_758_MOESM1_ESM.pdf]
