# Supplementary material for: Preserved skeletal muscle protein anabolic response to acute exercise and protein intake in well-treated rheumatoid arthritis patients
Source: Arthritis Res Ther. 2015 Sep 25;17:271. doi: 10.1186/s13075-015-0758-3 (PMC4583143; doi:10.1186/s13075-015-0758-3)

Additional file 3. mRNA expression at baseline

Gene expression data were normalized to RPLP0, log-transformed for statistical analyses and shown on a logarithmic scale as geometric mean ±SEM. Baseline expression of all targets is expressed relative to mean Healthy at baseline. Black bars denote rheumatoid arthritis patients (RA, n=13) and grey bars healthy controls (CON, n=13). No significant differences were observed between groups.

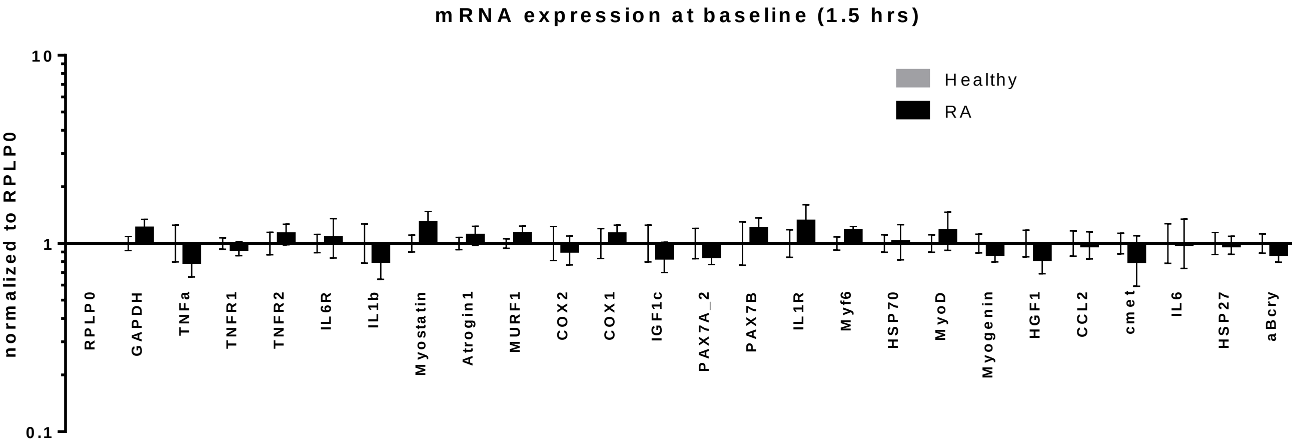

Supplement: Additional file 2: — mRNA expression at baseline. Baseline expression of all targets is expressed relative to mean healthy CON at baseline. Gene expression data were normalized to RPLP0, log-transformed for statistical analyses and shown on a logarithmic scale as geometric mean ± SEM. Black bars denote rheumatoid arthritis patients (RA, n = 13) and grey bars healthy controls (CON, n = 13). No significant differences were observed between groups. (PDF 177 kb) [file 13075_2015_758_MOESM2_ESM.pdf]
