# Supplementary material for: Preserved skeletal muscle protein anabolic response to acute exercise and protein intake in well-treated rheumatoid arthritis patients
Source: Arthritis Res Ther. 2015 Sep 25;17:271. doi: 10.1186/s13075-015-0758-3 (PMC4583143; doi:10.1186/s13075-015-0758-3)
Supplement: Additional file 3: — Comorbidities. Table of reported comorbidities in rheumatoid arthritis patients (RA) and healthy controls (CON). The number of subjects reporting each comorbidity is given. (PDF 179 kb) [file 13075_2015_758_MOESM3_ESM.pdf]

Additional file 1. Comorbidities

**Table S1**	**Comorbidities**	
	**CON**	**RA**
Allergy	0	2
Apopleksy	1	0
Asthma	0	4
Cancer	1	0
Cataract	0	1
COPD	0	1
Depression	0	1
Diverticulosis coli	0	1
Epilepsy	0	1
Fibromyalgia	0	1
Gastric ulcer	0	1
Hemorrhoids	0	1
Herpes	1	0
Hyper-cholesterolemia	1	0
Hypertension	2	3
Ichthyosis	0	1
Osteoporosis	0	1
Prostatic hyperplasia	0	1
Shingles	0	1
Slipped disc	1	1
Struma	0	1
VitB12 uptake deficiency	0	1
**No co-morbidity**	**7**	**2**
